# Pathological and immunological differences of arterial thrombi and wall caused by three different periodontal bacterial injections in rat models and proposals on the pathogeneses of vascular diseases

**DOI:** 10.1002/cre2.391

**Published:** 2021-01-18

**Authors:** Takehisa Iwai, Yoshiki Matsui, Kaori Homma, Tamiko Takemura, Mutsunori Fujiwara, Norio Aoyama, Asuka Furukawa, Hiroki Sato, Yuichi Izumi

**Affiliations:** ^1^ Division of Vascular Surgery and Collagen Disease Medicine Tsukuba Vascular Center Moriya Japan; ^2^ Section of Pathology Japanese Red‐Cross Medical Center Tokyo Japan; ^3^ Kanagawa Dental University Hospital Yokosuka Japan; ^4^ Department of Human Pathology Tokyo Medical and Dental University Tokyo Japan; ^5^ Department of Periodontology Tokyo Medical and Dental University Tokyo Japan

**Keywords:** arterial thrombus, rat models, three species of periodontal bacterial injection, thromboangiitis

## Abstract

**Objectives:**

Periodontal bacteria that have been studied show a strong connection to various vascular diseases. Among the many kinds of periodontal bacteria, *Porphyromonas gingivalis* (*Pg*) is well examined in the general aspects and in a rat model. However, whether other periodontal bacteria work or react differently is not studied well.

**Material and methods:**

We chose *Aggregatibacter actinomycetemcomitans* (*Aa*) and *Prevotella intermedia* (*Pi*) as different types of periodontal bacteria. Low‐density and high‐density bacterial solutions were injected in the small artery of rats' groins using our rat model. Eighteen limbs of 9 SD male rats (500–650 g) were used. After 7 days, 14–18 days, and 28 days, the rats were sacrificed. A pathological and an immuno‐histochemical study was conducted and reported on the low‐density group with 12 limbs because the *Pi* group lacked a high‐density study. Immuno‐histochemical staining of live *Pg* was performed on three limbs of three rats at 1 h, 3 h, and 1 week after injection.

**Results:**

The appearances from the acute, at 7 days, to chronic phases, at 28 days, were observed. The differences of the species were certainly observed in the internal elastic lamina (IEL), and immuno‐histochemical reactions. The inflammatory reactions, such as cellular distribution or intra‐thrombus materials, were similar in all. One week later, we could not see any living bacteria in the specimen or immunological observation.

**Conclusions:**

The three species were essentially the same, except for *Aa*'s stronger disruption of IEL, and more CD3 (Pan T cell) in *Pi* and more CD79a (Pan B cell) in *Pg*. We propose a new concept of a possible mechanism of vascular diseases, in which the work of LPS (lipopolysaccharides) and a toll‐like receptor (TLR) is emphasized.

## 1 INTRODUCTION

*Porphyromonas gingivalis* (*Pg*) is well and widely examined (Li et al., 2002; Li et al., 2008; Mikuls et al., 2014). The relationship between periodontal bacteria, especially *Pg*, and arterial lesions of the abdominal aorta (Kurihara et al., 2004), the arteries of the extremities (Iwai et al., 2005), and the cervical arteries (Wu et al., 2000) were reported from a mostly positive or causative standpoint. Periodontal bacteremia (Carroll & Sebor, 1980; Lockhart et al., 2008) is a common occurrence in patients suffering from chronic periodontitis or gingivitis; however, the properties of the periodontal bacteria‐containing arterial thrombi are not reported, because of the absence of good animal models prior to our recent report (Iwai et al., 2019). In our report, the thrombus model that employed *Pg* demonstrated pathological and immuno‐histochemical aspects of experimental thrombus formation. *Pg* injected vessels showed an acute inflammatory reaction followed by chronic fibrous changes in only 28 days. Immunohistochemistry showed the expected inflammatory changes induced by noninvasive bacteria *Pg*. These immuno‐histochemical changes seemed to be a natural immunological response.

Furthermore, pathogenic bacteria are counted in about 10 species (Socransky & Haffajee, 2000) and the differences of pathological action in the other bacteria are unclear. The *Pg* are indwelling in the blood stream, in a clotted platelet (Li et al., 2008) or in a monocyte itself (Suwatanapongched et al., 2010), and might have died within hours, even though they arrived alive, because they are non‐invasive and anaerobic and show pathologically low toxicity outside the gingival pocket of dental biofilms (Offenbacher et al., 2008). They died in the thrombus leaving their DNA or toxic material, such as lipopolysaccharides. As for *Aggregatibacter actinomycetemcomitans* (*Aa*) bacteria, some reports show their presence in heart disease or thoracic aortic lesions (Ishihara et al., 2004), but *Pg* bacteria were found in the extremities' vascular lesions or mid‐sized arteries and varices (Iwai et al., 2005; Seymour et al., 2007; Wu et al., 2000). These differences may be clear from the pathological or toxicological differences in our study.

This time, three species of the periodontal bacteria, *Pg*, *Aa*, and *Prevotella intermedia* (*Pi*), were used in the same densities and almost the same amounts of bacterial volume due to vessel thickness. We chose these two species because they were the ones obtained from the occluded Buerger disease patients' arteries (Iwai et al., 2005). *Aa* is believed to be the specific virulent species among periodontal bacteria and has been discovered in coronary or thoracic aortic specimens of atherosclerosis (Ohki et al., 2012). In Buerger disease cases, the most prevalent species was *Treponema denticola* (*Td*). However, we were unable to use it, because we lack a culture system for it in the Periodontology Department. The main purpose is to learn whether the three species are also in the same group as oral bacteria showing similar action and activity for the thrombus formation and immunological reaction.

**FIGURE 1:**
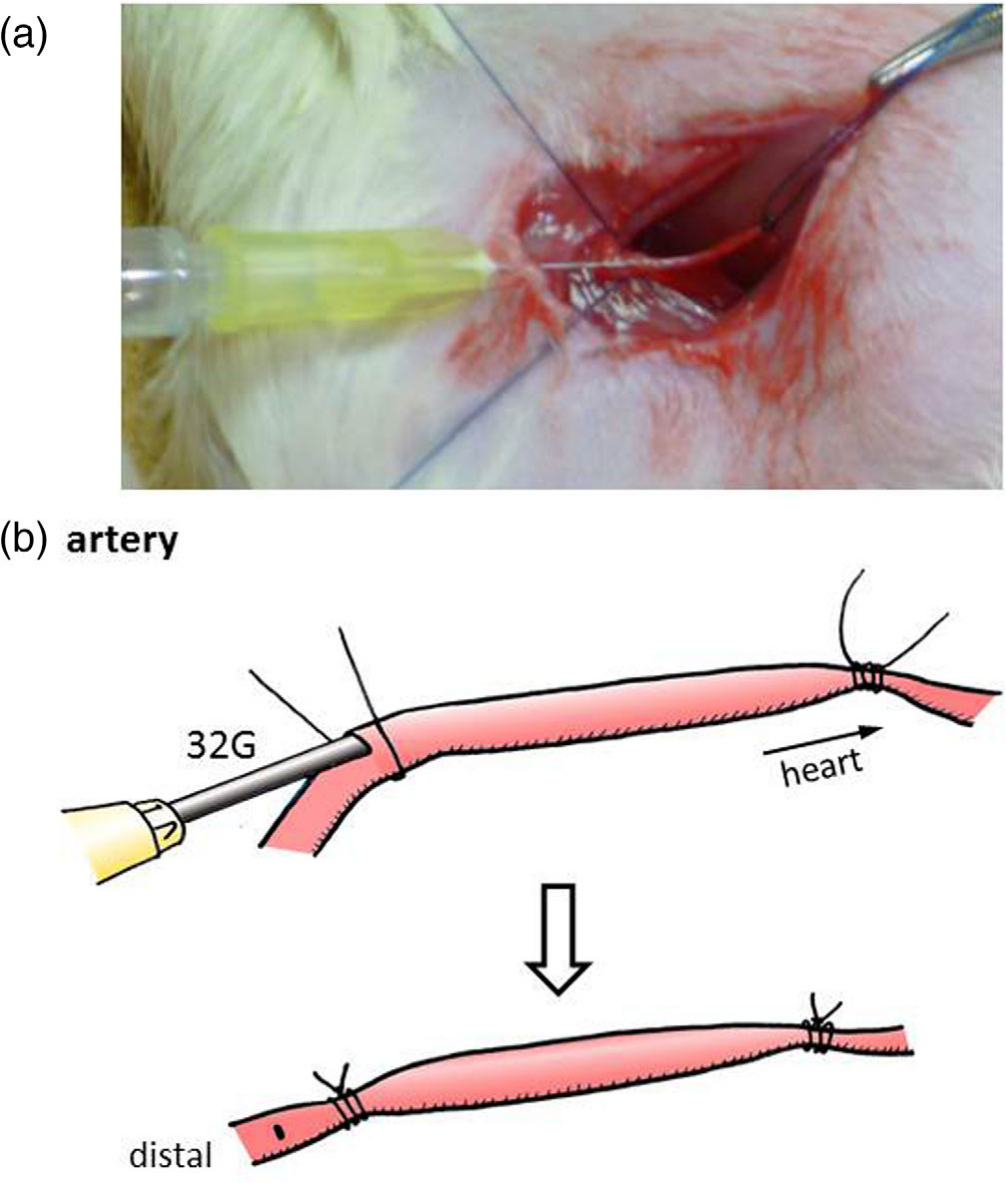
(a) Arterial puncture of the *Pg*, *Aa*, *Pi*, and control solutions in the rat. (b) Technical method of the injection into the artery (Iwai et al., 2019)

Actually, the loose ligation method, in which sausage‐like shape vessels are made for bacterial injection, was employed. Arterial thickness (0.6–1.0 mm in diameter) might be an associated factor, both technically and medically (Figure 1). Each periodontal bacterium seems to have a different nature or toxic reaction to the thrombus and arterial wall. In our rat model, the groin artery was thought to be the smallest one that was possible to inject without incurring distal tissue loss. Other methods include the stenosis formation method or temporary tight ligation that is then released after several hours. However, in these, the thrombus formation rate is low or complicated in an aseptic condition.

In the final session of this article, we added possible pathogeneses and mechanisms of vascular diseases such as thromboangiitis obliterans (Buerger disease) and atherosclerosis.

## 2 MATERIALS AND METHODS

SD male rats of 500–650 g body weight were used. All the rats were healthy without hypertension, diabetes, or hyperlipidemia (dyslipidemia). In all, 18 limbs of 9 rats were injected with bacteria and phosphate buffer saline (PBS) as controls. General anesthesia and aseptic maneuvers were used. The groin incision revealed small arteries, about 1 mm in diameter, and exposed 10–15 mm long arteries. The proximal and distal artery areas were ligated loosely with 4–0 monofilament nylon suture. Then, using a 32‐gauge needle, periodontal bacteria *Pg* (strain: A7A1‐28), *Aa* (strain: Y4), and *Pi* (strain: ATCC25611) of the same densities were injected; about 0.3 ml for vessels (Figure 1). The injected solutions were PBS as a control without bacteria, and a low and high densities of 1 × 10^7^ and 1 × 10^8^ CFU (colony‐forming units)/ml of *Pg*, *Aa*, and *Pi* bacteria solution. The surrounding area was washed well with normal saline and the wound was closed carefully. Postoperatively, no antibiotics were used. The weight of the rats was measured every day. The method of bacterial culture, *Pg* and *Aa*, is described in our co‐author's paper (Aoyama et al., 2011). A *Pi* culture was done following the *Pg* method.

Postoperatively, 7, 14–18, and 28 days were selected for study following their sacrifice. The specimens were fixed with a 10% formalin solution or a 4% paraformaldehyde phosphate buffer solution, and embedded in paraffin as usual. The slices, which were from the proximal one‐half of the specimen, were stained mainly with Hematoxylin Eosin (HE) and Elastica van Gieson (EVG). Part of the sliced specimen was examined using smooth muscle cell staining. Components of the blood cells of all the arterial thrombi, including the vessel walls, were analyzed as much as possible by the pathologist using a blind method. The cells and materials included red blood cells (RBC), granulocytes, the usual lymphocytes, plasma cells, macrophages (monocytes), platelets, giants cells, elastic fibers, hemosiderin, fibrin, and recanalization, including their quantitative grades (1): less than 10 cells, (2): 11–20 cells, (3): 21 cells, or (1): few, (2): moderate, (3): rich for a non‐cell material. The IEL was classified by disruption, shrinkage, and reduplication. CD3 (Pan T cells) and CD79a (Pan B cells) were immunologically stained and their location and quality were examined. Finally, we chose low‐density groups, 12 limbs, because the *Pi* group lacked a high‐density specimen and no significant differences between *Pg* and *Aa* in the two densities. As for the live study, three rats were sacrificed for 1 h, 3 h, and 1 week, respectively.

Cell or material distribution in the thrombus, and the occluded vessel was evaluated using the Student's *t* test to compare the groups.

An inter‐leukin‐6 (IL‐6) immuno‐histochemical study was carried out in the three groups and control specimens.

Observation of live *Pg* using immuno‐histochemical staining (Rajakaruna et al., 2018) was performed separately from three pieces in the study using three rats (Figure 4).

### 2.1 Statement on ethical approval

The animal experimentation was conducted according to the protocol approved by the director of the Moriya Keiyu Hospital after the review by Institutional Animal Care and Use Committee.

- Ethic committee Permit No. RO2‐11.
- Institutional Animal Care and Use Committee Permit No. SKL‐A‐13063/SKL‐A14020/SKL‐A‐14029/SKL‐A‐15014/SKL‐A‐15072/SKL‐A‐16010.

This experiment is compliant with the following guides:

- Act on Welfare and Management of Animals (October 1, 1973; Law No. 150).
- Guidelines for Proper Conduct of Animal Experiments (Science Council of Japan).

## 3 RESULTS

**FIGURE 2:**
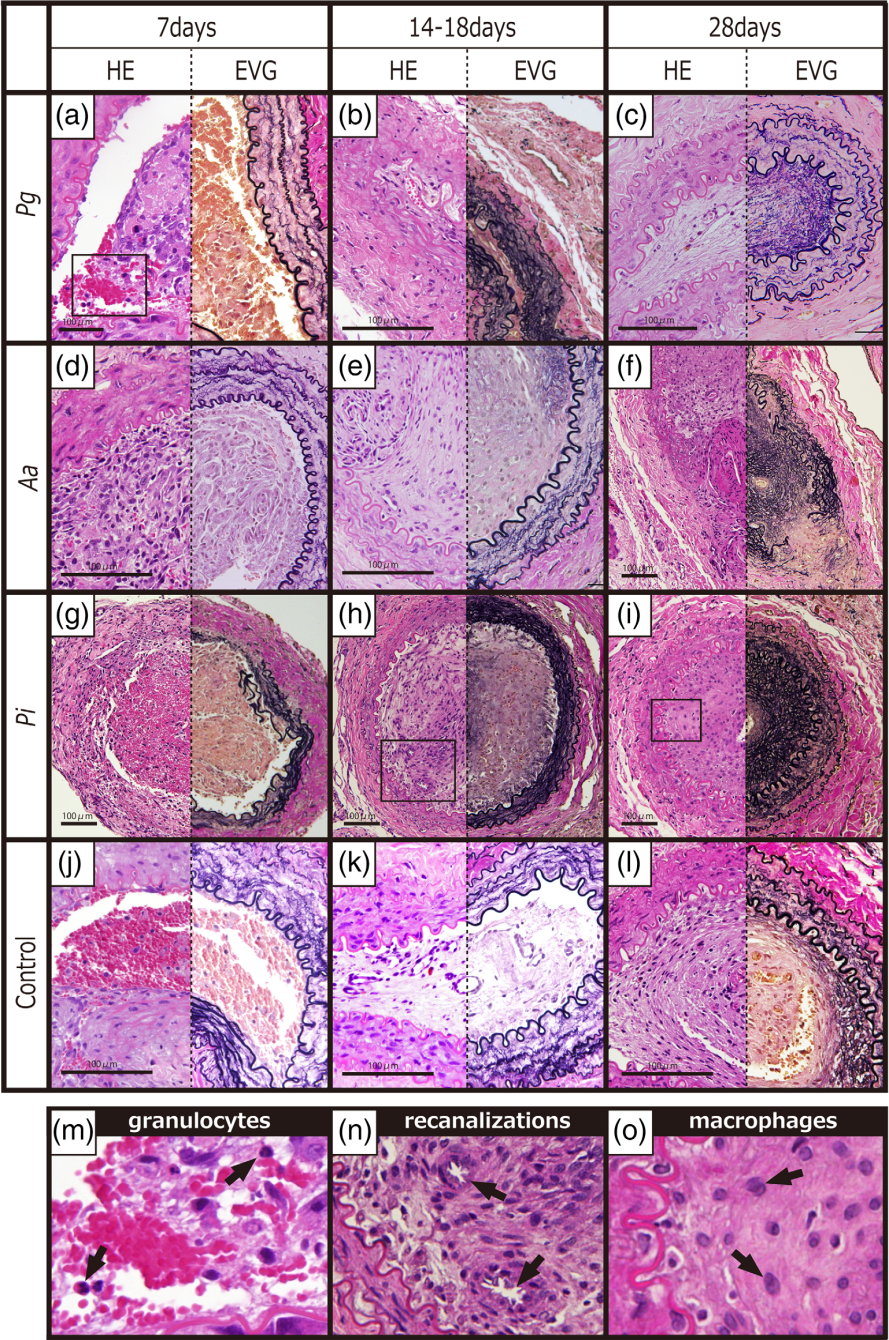
Pathological serial changes after injection of *Pg*, *Aa*, and *Pi* to the artery and the control. (a) 7 days after *Pg* 1 × 10^7^ CFU/ml injection (left HE, right EVG stain ×400); (b) 14–18 days after the same *Pg* injection (left HE stain ×400, right EVG stain ×100); (c) 28 days after the same *Pg* injection (EVG&HE stain ×100); (d) 7 days after *Aa* (left) (HE stain ×100); (e) 14–18 days after the same density *Aa* injection (HE stain ×400); (f) 28 days after same *Aa* injection (HE stain ×100); (g) 7 days after *Pi* injection (HE and EVG stain ×200); (h) 14–18 days after *Pi* injection (HE and EVG stain ×200); (i) 28 days after the same *Pi* injection (HE and EVG stain ×200); (j–l) pictures of control show no granulocytes and the intact IEL; (m–o) enlarged views of granulocytes (from a), recanalization (from h) and macrophages (from i) in the figure

All the rats were alive, the wounds were clear without infection, and no limb necrosis was observed. The weight of the rats gradually increased and no significant change between the control rats and injected rats was seen. All specimens were obtained using sterile techniques. The results showed the ligation site was adequate. Animal care was satisfied. The rat's ligation site was the external iliac artery, which does not cause limb necrosis, even in human cases. Thrombus formation was observed in all specimens including the control group. At 7 days after the *Pg* 1 × 10^7^ injection, microscopic examination revealed (Figure 2) many red blood cells, moderate granulocytes, a few lymphocytes, macrophages, and spindle cells in the thrombus. The IEL was intact. In the media, granulocytes and edema were present. At 14–18 days after the same *Pg* injection, the granulocytes disappeared and recanalization appeared in the thrombus. The IEL showed disruption, shrinkage, and reduplication. Edematous media and outer elastic lamia disruption were present. At 28 days after the same *Pg* injection, lymphocytes and plasma cells were present in the thrombus. Elastic fibers were numerous. The IEL showed shrinkage. The media revealed minimum changes. At 7 days after *Aa*, many granulocytes were present in the fresh thrombus. The IEL was intact. The media showed a few granulocytes and edema. At 14–18 days after the same‐density *Aa* injection, elastic fibers were rich in the thrombus. The IEL showed disruption and shrinkage. At 28 days after the *Aa* injection, plasma cells were moderately rich in the thrombus. Giant cells were outside of the adventitia. The IEL showed disruption and shrinkage with reduplication. At 7 days after *Pi* injection, there were a few RBC, granulocytes, lymphocytes, and rich macrophages in the thrombus. The IEL was intact. At 14–18 days after the *Pi* injection, moderate granulocytes, macrophages, and spindle cells were present in the thrombus. At 28 days after the same *Pi* injection, there were rich spindle cells, and lymphocytes in the thrombus. The IEL showed disruption, shrinkage, reduplication and moderate rupture. In the thrombus, marked fibrous changes were present. Pictures of the control showed no granulocytes and the intact IEL. No bacterium was seen in any phase in microscopic observation and immuno‐histochemical staining. The other changes are shown in Table 1A.

**TABLE 1:** (A) HE and EVG staining result of the *Pg*, *Aa*, and *Pi* injection; (B) immunohistochemistry of the arterial specimen *Pg*, *Aa*, and *Pi*

			Control group (*n* = 3)			*Pg* group (*n* = 3)			*Aa* group (*n* = 3)			*Pi* group (*n* = 3)		
		Days	7	14–18	28	7	14–18	28	7	14–18	28	7	14–18	28
A	Thrombus	RBC	2	0	1	3	0	0	2	1	0	3	0	0
		Granulocyte	0	0	0	2	0	0	1	0	0	1	1	0
		Lymphocyte	1	2	1	1	1	1	1	1	0	3	3	1
		Plasma cell	0	0	0	0	0	1	0	0	0	1	0	0
		Macrophage	1	1	1	1	1	1	1	0	0	2	1	1
		Recanalization	0	2	1	0	2	1	1	2	3	1	2	1
		Elastic fiber	1	1	1	0	1	3	2	3	1	0	2	3
	Internal elastic lamina	Disruption	0	0	0	0	1	0	3	2	1	1	1	0
		Shrinkage	0	0	0	0	2	1	0	1	3	0	0	0
		Reduplication	0	0	0	0	2	0	0	0	1	0	0	0
	Media	Granulocyte	0	0	0	1	0	0	0	0	0	2	1	0
		Lymphocyte	0	0	0	1	1	0	1	0	0	1	1	0
		Plasma cell	0	0	0	0	0	0	0	0	0	0	0	0
		Swelling	1	1	1	1	1	1	1	0	1	1	1	1
B	CD3 (Pan T cell)		0	1	1	2	1	1	1	1	1	2	2	2
	CD79a (Pan B cell)		0	1	0	2	2		0	1	1	1	1	1

*Notes*: Control: phosphate buffered saline. Quantitative count criteria of blood cells: RBC (red blood cells), granulocytes, lymphocytes, plasma cells, macrophages: less than 10 cells (1), 11–19 cells (2), 20 cells (3). Elastic fiber, internal elastic lamina (IEL) (disruption, shrinkage, reduplication) and recanalization classification: none (0), few (1), moderate (2), rich (3). Count criteria: see Figure 3.

**FIGURE 3:**
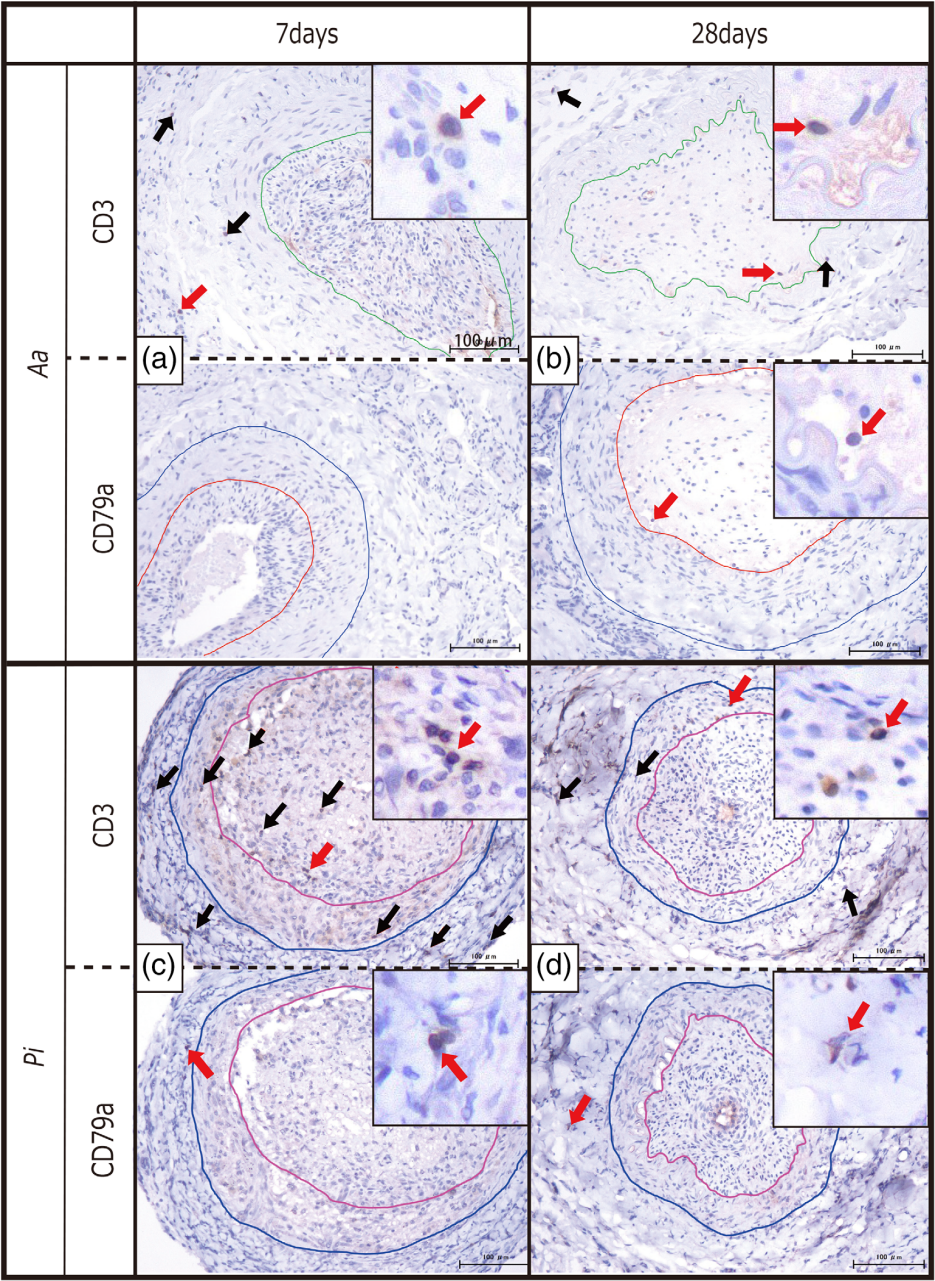
Immuno‐histochemical staining of *Aa* and *Pi*. CD3 (Pan T cell) and CD79a (Pan B cell) at 7 and 28 days are shown. *Pg* findings are in the literature (Iwai et al., 2019). Red arrows are positive cells. In the corner, the enlarged ones are added. (a) *Aa* at 7 days, CD3 (1) in the surrounding area and no reaction in the thrombus. CD79a did not show in the entire area. (b) At 28 days, CD3 was shown with arrows. CD79a (1) in the thrombus with an arrow. (c) *Pi* at 7 days, CD3 (2) in the thrombus and surrounding area. CD79a (1) in the surrounding area. (d) At 28 days, CD3 (2) in the thrombus, the media and surrounding area. CD79a (0) in the thrombus and the media, (1) surrounding area. Quantitative criteria: less than 10 cells (1), 11–19 cells (2), more than 20 cells (3)

Immuno‐histochemical staining showed the same amount (1–2) of CD3 (Pan T cell) and CD79a (Pan B cell) cells in *Pg* and less in the *Aa* groups. The *Pi* group showed that CD3 was maintained moderately (2) during the three phases and a smaller amount (1) of CD79a cells. These cells were seen in the control groups (0) or (1) (Figure 3, Table 1B).

Statistical analysis revealed (a) cells and materials changes in the thrombus were not significant between three groups, (b) disruption of the IEL was significantly (<0.05) remarkable in the *Aa* group, (c) CD3 (Pan T cells) positive cells were significantly (<0.05) rich in the *Pi* group, and (d) CD79a (Pan B cells) were significantly (<0.05) rich in the *Pg* group.

**FIGURE 4:**
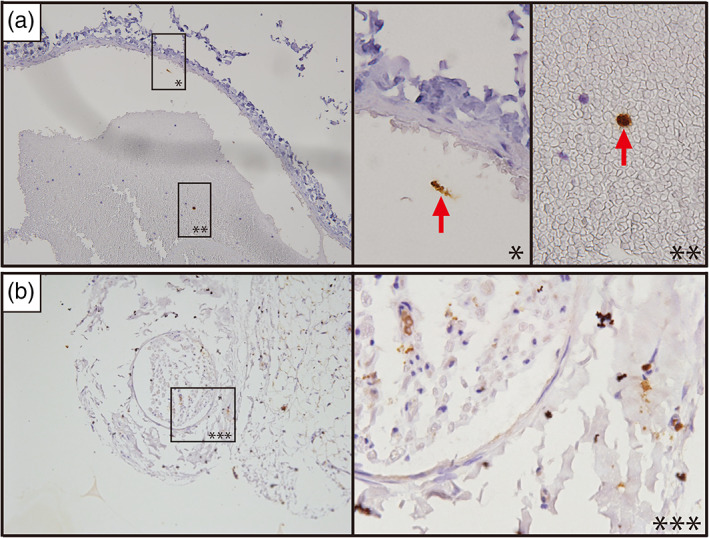
Immuno‐histochemical staining of live *P. gingivalis*. (a) 3 h after injection of *Pg*. Two positive staining bacteria (red arrow) were observed. (b) After 1 week, no positive bacteria were shown

Using an immune‐histochemical staining technique (Rajakaruna et al., 2018), our observation of live *Pg* suggested it lives several hours maintaining its shape after causing a thrombus in the rat (Figure 4a). One week later we could not see any living bacteria in the specimen or immunological observation (Figure 4b). With this technique, it is impossible for the bacteria to be washed out during the process. An IL‐6 study was carried out. However, because of the timing or other reasons, good pictures were not obtained.

## 4 DISCUSSION

The thrombosis animal model of bacterial exposure has been shown in several methods. Only with the stenosis technique, it is very difficult to form thrombosis by bacteria injection; other methods use tight ligation in the proximal and distal arteries and then inject the bacteria to reopen the wound to release the ligatures completely several hours or days later (Nagase, 1975). This method is quite troublesome. Our method formed the luminal thrombus in all specimens. The early development of recanalization in the arterial model seems to mean that, in the ligation, the slippery material that is used is loose enough to allow a small flow from the proximal to the distal lumen. It is a standard technique for thrombus formation study (Iwai et al., 2019).

The density of bacteria is another problem, so from the two kinds of standard densities for animal experiences, we chose the weaker one based on the academic report (Genco et al., 1991). In our previous report, we chose both densities and reported the negligible differences with *Pg* (Iwai et al., 2019). Therefore, the density which we chose here was reasonable for this animal study. The pure cultured bacteria, *Pg*, *Aa*, and *Pi*, were obtained from the Laboratory of the Periodontal Department, Tokyo Medical and Dental University, which has a number of academic reports on periodontal bacteriology.

Of course, the study might be done by poly‐microbial injection in the next step because of the poly‐microbial nature of periodontal disease. However, as the first step, it will be necessary to know the reaction of each one of the species.

**FIGURE 5:**
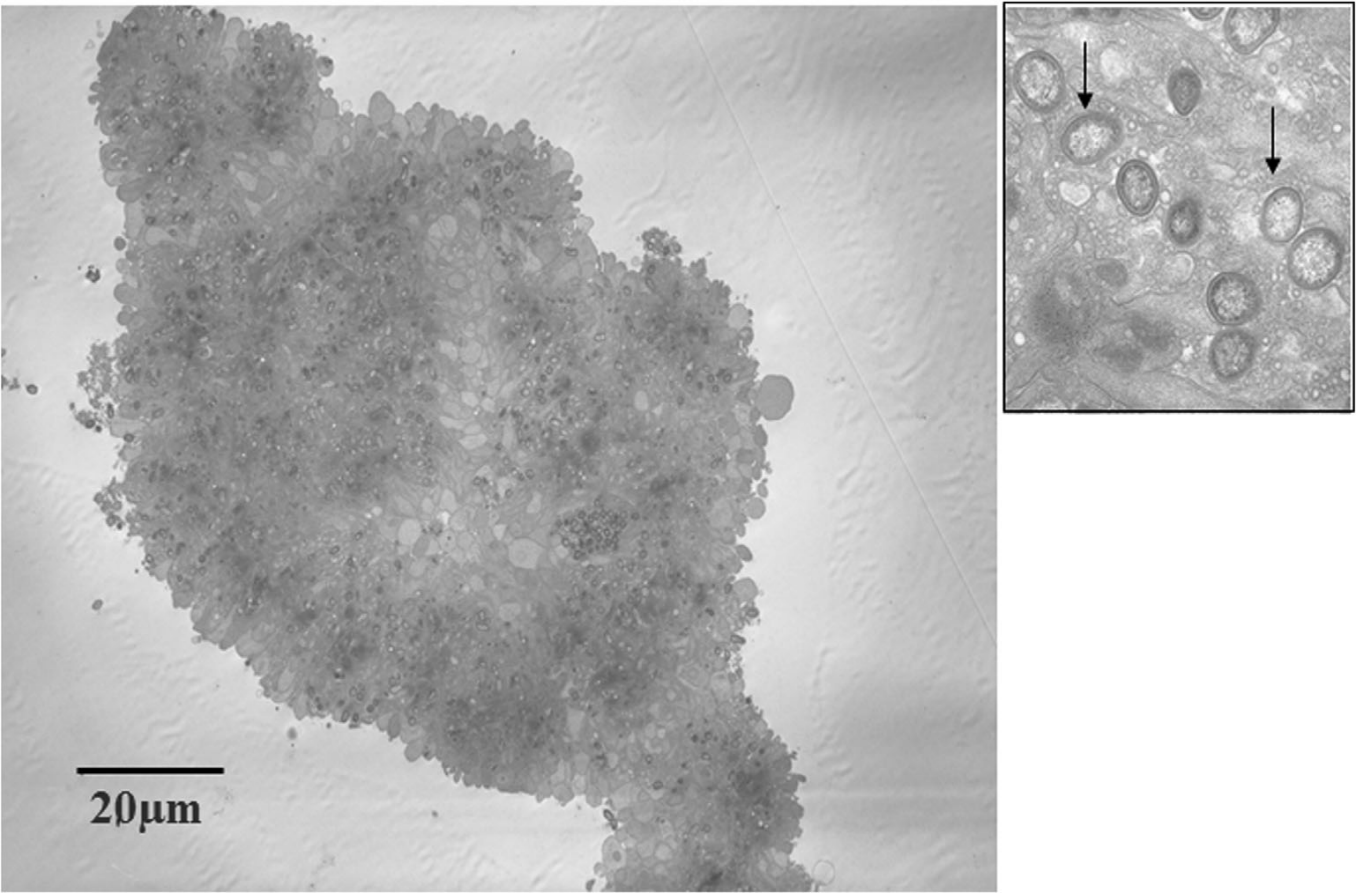
Platelets aggregated mass showing those over 100 μm in diameter. *Pg* is indwelling inside of the platelets (Li et al., 2008). The arrow shows *Pg* bacteria in a great number of aggregated platelets. This is a phase contrast electron microscopic view

The length of the observation was decided from the increased titer of the immunological reaction in the rat model since 28 days is almost sufficient (Harada, 2002). The rat's weight increased at the same rate as that of the control rats. This means that the whole body changes were not caused by an infection. The main transportation system of *Pg* seems to be achieved by the mass production of platelets (Li et al., 2008) (Figure 5); these masses will then occlude the very small arteries. On the other hand, *Pg* is transferred indwelling in the monocytes (Suwatanapongched et al., 2010), which promotes adhesion to the luminal wall in combination with adhesion factors. The above‐mentioned study is limited to *Pg* bacteria, but almost the same mechanisms may be used on the *Aa* and *Pi* bacteria as they are in the same category of bacteria. Other possible paths to enter the vessels may be discussed. In vitro, *Pg* has been reported to invade and survive in human oral cells, including buccal epithelium cells (Rudney et al., 2001), oral epithelium cells (Sandros et al., 1993), and gingival epithelium cells (Lamont et al., 1995), as well as in human coronary artery cells (Dorn et al., 1999). These facts are interesting in terms of the character of the periodontal bacteria.

Due to its several limitations, our small arterial rat model seems to be a useful model in which it is possible to observe actual inflammatory thrombus and arterial conditions using a microscope.

The thrombus showed acute inflammatory changes with granulocytes (neutrophils) at 7 days after injection and settled the condition at 28 days, showing a fibrous cell increase. The direct bacterial injection might induce the innate immunological reaction showing the macrophages or plasma cells that appeared in our most recent specimen and immuno‐histochemical staining study. In practical medicine, the bacteremia caused by periodontal bacteria will continue for a long period and will show aseptic inflammation within the thrombus and vessels walls. This condition was observed in the *Pg*, *Aa*, and *Pi* groups in almost the same fashion. Unfortunately, IL‐6 immuno‐histochemical staining was not successful this time. It will be re‐done using new raw materials. These pathological phenomena are strongly suggestive of the same pathology of thromboangiitis (thrombitis plus angiitis) in which we found the *Pg*, *Td*, or *Pi* bacterial DNA in Buerger disease patients. No proliferation of the bacteria in the specimens will mean that toxic bacterial components, lipopolysaccharides, etc., may react in the thrombus in the *Pg*, *Aa*, and *Pi* groups equally, so the blood chemistry changes are minimal from a clinical perspective, except for a slight elevation in the highly sensitive CRP (C‐reactive protein) level in clinical cases (Noack et al., 2001; Olin, 2000). A very recent supportive research report showed that the serum levels of the toll‐like receptor 4 were significantly higher in the acute phase of thromboangiitis patients, which indicates the trigger of the disease might be Gram‐negative bacteria, such as those in our groups, *Pg*, *Aa*, or *Pi* (Mohareri et al., 2018). Another report on Buerger disease that studied the toll‐like receptor, suggested the presence of innate immunity (Koizumi et al., 2019), which was demonstrated in our animal models.

The differences among the bacterial species or amounts were estimated by the changes of granulocyte counts, lymphocyte counts, or the disruption of the internal elastic lamina. *Aa* had an apparently strong reaction to the IEL, showing severe disruption (<0.05). The *Pi* group showed a different reaction in the cellular numbers shown in Table 1A, but the fibrous reaction was rich compared with the other two groups. The granulocytes' invasion to the media were shown in three groups and the *Pi* group demonstrated more invasion (Table 1A). These rat model results seem to reflect the clinical course of oral bacteria‐related‐diseases.

The immunological staining in the three groups showed almost the same reaction, demonstrating moderate increases of T cells and B cells, which indicated the progression of an innate immunological reaction. Also, it seemed to be in preparation for the acquired immunological reaction. Among the three groups, *Pg* and *Pi* seem to have had a slightly stronger immunological reaction as shown in the results. The histological observation in all three groups was mainly thrombitis and partly angiitis, as shown in Table 1A. The only differences between *Pg* and *Aa* were the destroy strength on the IEL and that *Pi* showed apparently richer fibrous changes in the thrombus and IEL area. Other well‐known species of periodontal bacteria may react the same, to a greater or lesser degree. The explanation of related diseases, such as low‐weight babies (Vergnes & Sixou, 2007) or rheumatoid arthritis (Mikuls et al., 2014), may require more examination using the rat models, and not only a pathological study, but also new technical analysis.

## 5 POSSIBLE MECHANISMS OF THE THROMBITIS, ANGIITIS, AND THROMBOANGIITIS FROM OUR RAT MODEL INDUCED BY THE PERIODONTAL BACTERIA, AND THE LITERATURE

It is clear that the periodontal bacteria (Gram‐negative bacteria) appeared and became fixed in the peripheral area of the artery or vein. At least three mechanisms act in the area. The first one is a micro‐embolic occlusion of the peripheral artery. It is apparent because the platelets aggregated mass was observed to reach up to 100 μm in diameter, which cannot pass through the capillary channel (Li et al., 2008). This occlusion gradually accumulates in the proximal area. The phase‐contract electron microscope showed the mass clearly after dropping the bacteria into the platelet rich suspension (Figure 5).

The second one is a reaction with the internal surface (endothelial cells) and the bacteria. LPS of the bacterial membrane break the endothelial cells and invade the IEL and the media (Bnnerman & Goldblim, 1999). In the case of *Pg*, gingipain will act in the same way to break the luminal surface (Baba et al., 2002). It causes angiitis, which consists of granulocytes, macrophages, or plasma cells. Disruption of the IEL may lead to easy passage through the inflammatory cells. The third one is the adhesion of the monocyte, which includes the bacteria, to the luminal lumen (Suwatanapongched et al., 2010). The adhesion area will be the site of invasion.

All reactions may be followed by a cytokine release. Again, LPS is a characteristic material of Gram‐negative anaerobic bacteria, like periodontal bacteria, and acts as a cell killer. LPS is a substance of the Gram‐negative bacterial outer layer and it shows the toxic effect or biological activities. It is strongly related to the mechanism of the innate immunological reaction. The action of LPS will accumulate to disrupt the endothelial barrier function mostly from the endotoxic action with cytokine, or enhancement of immunity (Bnnerman & Goldblim, 1999; Meyrick et al., 1989; Penn & Chisolm, 1991). Recently the toll‐like receptor was studied and a relation with LPS reaction was found to impair the endothelial function (Zimmer et al., 2011). Using an immuno‐histochemical staining technique (Rajakaruna et al., 2018), our observation of *Pg* suggested it lived several hours, maintaining its shape after causing a thrombus in the rat (Figure 4a). At least, 1 week later we could not see any living bacteria in the specimen or immunological observation (Figure 4b). An old report (1975) showed Buerger disease‐like pathological changes after injecting LPS purified from *Escherichia coli* in a rabbit neck artery (Nagase, 1975). This suggests the same response in our rat model (Iwai et al., 2019).

**FIGURE 6:**
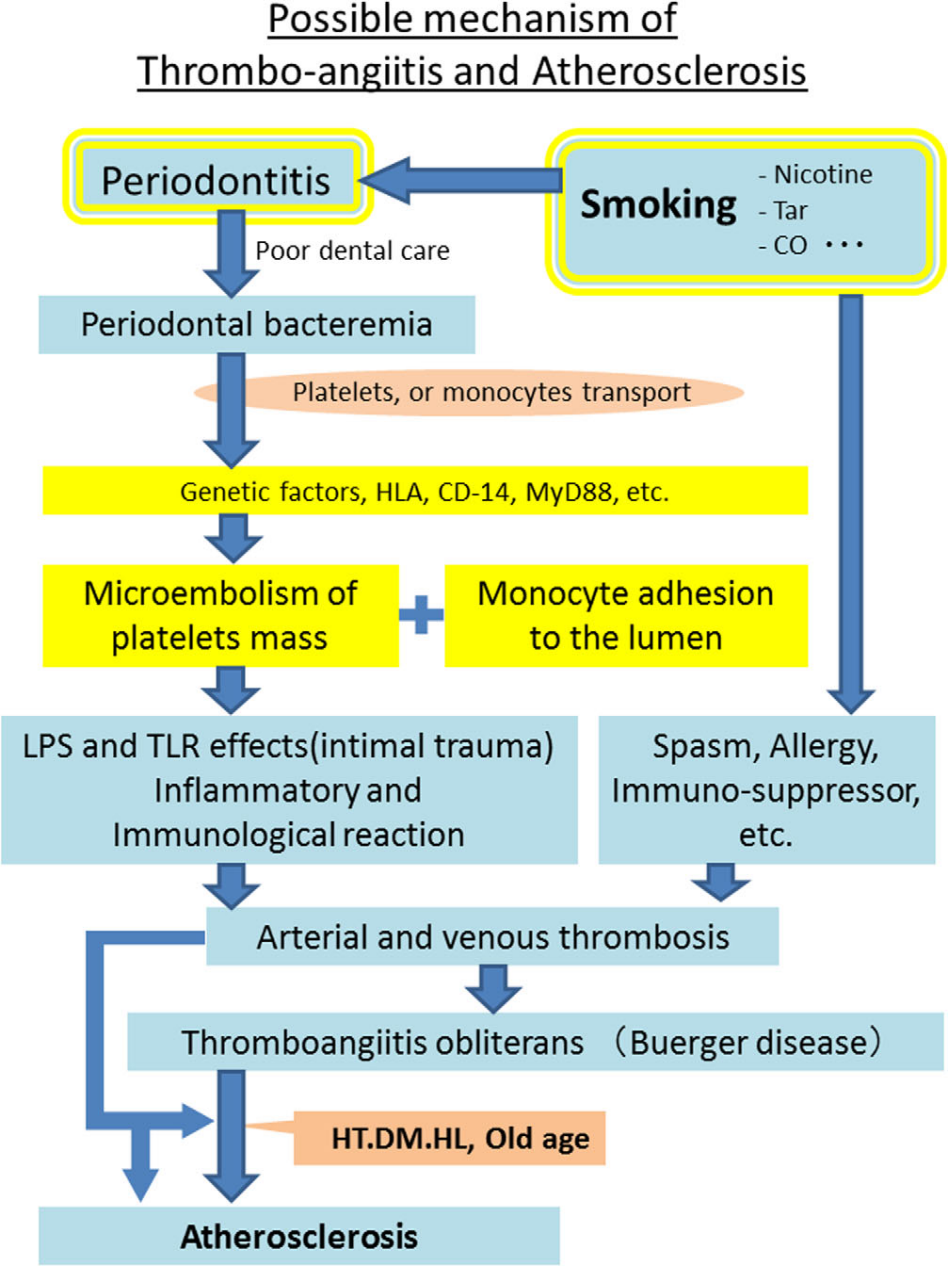
Possible mechanisms of thromboangiitis and atherosclerosis. DM, diabetes mellitus; HL, hyperlipidemia (dyslipidemia); HT, hypertension; LPS, lipopolysaccharides of Gram‐negative bacteria; TLR, toll‐like receptor

All of the possible mechanisms of thromboangiitis and related atherosclerotic changes are shown in Figure 6. This shows the combination mechanism with periodontal bacteremia and nicotine mixed with gene effects or bad oral care, etc. Finally, as a result of aging, thromboangiitis will change to atherosclerotic pathology modified by hypertension, hyperlipidemia (dyslipidemia), or diabetes mellitus, which are excluded from the strict exclusion criteria of the thromboangiitis (Buerger disease) diagnosis (Shionoya, 1990).

Buerger disease was discussed from 1957 to 1970 (Baker, 1962; Wessler et al., 1961) but there was confusion about whether it was just due to atheromatous changes in the young people. At that time, the combined changes, namely Buerger disease plus atherosclerosis, were accepted even in the case of pure Buerger disease. So, Buerger disease criteria excluded atherosclerosis‐accelerating factors such as hypertension, hyperlipidemia, and diabetes mellitus, except cigarette smoking (Shionoya, 1990). However when any factor is added to Buerger disease criteria, the pathological changes are not described. In 1998, Prof. Tanaka, a well‐known Japanese pathologist in angiology, proposed that Buerger disease and atherosclerosis have very similar processes (Tanaka, 1998). Buerger disease is a relatively pure change that occurs in young people who only smoke heavily and in unknown intimal trauma. Atherosclerosis is a disease complicated by smoking, hypertension, hyperlipidemia or diabetes mellitus, and unknown intimal trauma. Prof. Tanaka suggested a common factor: intimal trauma is the key to Buerger disease and atherosclerosis pathology and etiology. This time we were able to find one of the triggers of intimal trauma. It is Gram‐negative bacterial infection including LPS, which is present in the outer layer of the bacterial cell. Also, the bacteria should be non‐invasive and anaerobic. The bacteria died shortly after arriving in the peripheral area of the vessel. These specific bacteria will be limited, such as the periodontal bacteria itself.

Clinically, a Buerger disease patient becomes an atherosclerotic patient after 50 years of age when the patient suffers from atherosclerotic factors. Smoking has a lot of effects on the etiology of both vascular diseases. Nicotine, tar, and carbon monoxide lead to immunological suppression, ischemic gingiva, tooth damage, or vessel spasms around the teeth (Georgia & Guthmiller, 2007; Sopori, 2002), etc. Genetic relations are reported, such as, HLA, CD14 (Chen et al., 2007), etc. Following the micro‐embolism of platelets mass (Figure 5) or monocyte adhesion to the lumen, direct intimal trauma by LPS or a toll‐like receptor, and inflammatory and immunological reaction proceed. Then arterial and venous thrombosis occurs. This is the story of Buerger disease, and afterward, when it is modified by hypertension, hyperlipidemia, or diabetes mellitus, atherosclerosis occurs (Figure 6).

## 6 CONCLUSION

The arterial thrombus model caused by periodontal bacteria in rats was studied pathologically, including immunological staining as the second continuation of the previous report (Iwai et al., 2019). Three anaerobic periodontal bacteria (*Pg*, which is well‐studied, and *Aa* and *Pi*, which are not well‐studied), were used in same densities and same thicknesses using our own rat model. The findings showed no proliferation of the bacteria at 7 days and a granulocyte rich‐inflammatory reaction. At 28 days, the reaction shows fibrous changes with a macrophage and plasma cell increase. Immuno‐histochemistry staining showed a moderate increase of CD3 (Pan T cells) and CD 79a (Pan B cells) after injection of the bacteria, proving an innate immunological response. The pathological differences were observed by species, showing a slightly different tendency: namely, *Aa* is stronger in destroying power than *Pg and Pi*, pathologically (<0.05). Thrombus' cells and materials had no significant changes (<0.05). Whether more popular species, such as *Td* or *Campylobacter rectus*, are almost the same or not is the next interest of the study. More investigation is required, including poly‐microbial injection.

Finally, we concluded the pathogeneses and possible mechanisms of thromboangiitis and atherosclerosis, emphasizing the LPS plus toll‐like receptor reaction to the vessel wall as a traumatic initial effect. The initial‐traumatic damage to the endothelial cells is the most mystifying factor in understanding thromboangiitis and atherosclerosis. Further investigation with more study of immunological cells, or proteases, or cytokines (again), will be the next step.

## CONFLICT OF INTEREST

None.

## AUTHOR CONTRIBUTIONS

Conception and design: T. Iwai, M. Fujiwara; Analysis and interpretation: T. Iwai, K. Homma, T. Takemura, Y. Izumi; Data collection: T. Iwai, A. Furukawa, N. Aoyama; Writing the article: T. Iwai; Critical revision of the article: T. Iwai, H. Sato, N. Aoyama; Final approval of the article: T. Iwai, Y. Izumi; Statistical analysis: Y. Matsui; Acquisition of funding: T. Iwai; Overall responsibility: T. Iwai.

## Data Availability

This is an open access article under the terms of the Creative Commons Attribution License, which permits use, distribution and reproduction in any medium, provided the original work is properly cited.

## References

[cre2391-bib-0001] Aoyama, N. , Suzuki, J. , Wang, D. , Ogawa, M. , Kobayashi, N. , Hanatani, T. , Takeuchi, Y. , Izumi, Y. , & Isobe, M. (2011). *Porphyromonas gingivalis* promote murine abdominal aortic aneurysms via matrix metalloproteinase‐2 induction. Journal of Periodontal Research, 46, 176–183. 21143481 10.1111/j.1600-0765.2010.01326.x

[cre2391-bib-0002] Baba, A. , Kadowaki, T. , Asano, T. , & Yamamoto, K. (2002). Roles for Arg‐ and Lys‐gingipains in the disruption of cytokine responses and loss of viability of human endothelial cells by *Porphyromonas gingivalis* infection. Biological Chemistry, 383, 1223–1230. 12437109 10.1515/BC.2002.135

[cre2391-bib-0003] Baker, N. W. (1962). The case for retention of the diagnostic category “thrombo‐angiitis obliterans”. Circulation, 25, 1–4. 13865027 10.1161/01.cir.25.1.1

[cre2391-bib-0004] Bnnerman, D. D. , & Goldblim, S. E. (1999). Direct effects of endotoxin on the endothelium: Barrier function and injury. Laboratory Investigation, 79, 1181–1199. 10532583

[cre2391-bib-0005] Carroll, G. , & Sebor, R. J. (1980). Dental flossing and its relationship to transient bacteremia. Journal of Periodontology, 51, 691–692. 6937641 10.1902/jop.1980.51.12.691

[cre2391-bib-0006] Chen, Z. , Takahashi, M. , Naruse, T. , Nakajima, T. , Chen, Y. W. , Inoue, Y. , Ishikawa, I. , Iwai, T. , & Kimura, A. (2007). Synergistic contribution of DC14 and HLA loci in the susceptibility to Buerger disease. Human Genetics, 122, 367–372. 10.1007/s00439-117-0408-1. 17653770

[cre2391-bib-0007] Dorn, B. R. , Dunn, W. A. , & Progulske‐Fox, A. (1999). Invasion of human coronary artery cells by periodontal pathogens. Infection and Immunity, 65, 5792–5798. 10.1128/iai.67.11.5792-5798.1999 PMC96956 10531230

[cre2391-bib-0008] Genco, C. A. , Cutler, C. W. , Kapcyznski, D. , Moloney, K. , & Arnold, R. R. (1991). A novel mouse model to study the virulence of and host response to *Porphyromonas* (Bacteroides) *gingivalis* . Infection and Immunity, 59, 1255–1263. 2004807 10.1128/iai.59.4.1255-1263.1991 PMC257836

[cre2391-bib-0009] Georgia, K. J. , & Guthmiller, J. M. (2007). The impact of cigarette smoking on periodontal disease and treatment. Periodontology, 44, 170–194. 10.1111/j.1600-0757.2007.00212.x 17474933

[cre2391-bib-0010] Harada, M. (2002). Immunoresponses of model mice infected with *Porphyromonas gingivalis* . Journal of Fukuoka Dental College, 29, 69–82.

[cre2391-bib-0011] Ishihara, K. , Nabuchi, A. , Ito, R. , Miyachi, K. , Kuramitsu, H. K. , & Okuda, K. (2004). Correlation between detection rates of periodontopathic bacterial DNA in carotid coronary stenotic artery plaque and in dental plaque samples. Journal of Clinical Microbiology, 42, 1313–1315. 15004106 10.1128/JCM.42.3.1313-1315.2004 PMC356820

[cre2391-bib-0012] Iwai, T. , Inoue, Y. , Umeda, M. , Huang, Y. , Kurihara, N. , Koike, M. , & Ishikawa, I. (2005). Oral bacteria in the occluded arteries of patients with Buerger disease. Journal of Vascular Surgery, 42, 107–115. 16012459 10.1016/j.jvs.2005.03.016

[cre2391-bib-0013] Iwai, T. , Matsui, Y. , Homma, K. , Takemura, T. , Fujiwara, M. , Aoyama, N. , Sato, H. , & Izumi, Y. (2019). Oral‐bacterial‐induced arterial and venous thrombus in rats: Pathological and immunological studies. Clinical and Experimental Dental Research, 1–8. 10.1002/cre2.215. PMC6820577 31687183

[cre2391-bib-0014] Koizumi, S. , Naruse, T. K. , & Kimura, A. (2019). A haplotype of toll‐like receptor 1 is associated with resistance to Buerger disease in Japanese. Major Histocompatibility Complex, 26, 189–194.

[cre2391-bib-0015] Kurihara, N. , Inoue, Y. , Iwai, T. , Umeda, M. , Huang, Y. , & Ishikawa, I. (2004). Detection and localization of periodontopathic bacteria in abdominal aortic aneurysms. European Journal of Vascular and Endovascular Surgery, 28, 553–558. 15465379 10.1016/j.ejvs.2004.08.010

[cre2391-bib-0016] Lamont, R. J. , Chan, A. , Belton, C. M. , Izutsu, K. T. , Vasel, D. , & Weinberg, A. (1995). *Porphyromonas gingivalis* invasion of gingival epithelial cells. Infection and Immunity, 63, 3878–3885. 7558295 10.1128/iai.63.10.3878-3885.1995 PMC173546

[cre2391-bib-0017] Li, L. , Messas, B. , Batista, E. I., Jr. , Levine, R. A. , & Amar, S. (2002). *Porphyromonas gingivalis* infection accelerates the progression of atherosclerosis in a heterozygous apolipoprotein E‐deficient murine model. Circulation, 105, 861–867. 11854128 10.1161/hc0702.104178

[cre2391-bib-0018] Li, X. , Iwai, T. , Nakamura, H. , Inoue, Y. , Chen, Y. , Umeda, M. , & Suzuki, H. (2008). An ultrastructural study of *Porphyromonas gingivalis*‐induced platelet aggregation. Thrombosis Research, 122, 810–819. 18448150 10.1016/j.thromres.2008.03.011

[cre2391-bib-0019] Lockhart, P. B. , Brennan, M. T. , Sasser, H. C. , Fox, P. C. , Paster, B. J. , & Bahrani‐Mougeot, F. K. (2008). Bacteremia associated with tooth brushing and dental extraction. Circulation, 117, 3118–3125. 18541739 10.1161/CIRCULATIONAHA.107.758524 PMC2746717

[cre2391-bib-0020] Meyrick, B. , Hoover, R. , Jones, M. R. , Berry, L. C., Jr. , & Brigham, K. (1989). In vitro effect of endotoxin on bovine and sheep lung microvascular and pulmonary artery endothelial cells. Journal of Cellular Physiology, 138, 165–174. 2642914 10.1002/jcp.1041380122

[cre2391-bib-0021] Mikuls, T. R. , Payne, J. B. , Yu, F. , Thiele, G. M. , Reynolds, R. J. , Cannon, G. W. , Marks, J. , McGowan, D. , Kerr, G. S. , Redman, R. S. , Reimond, A. , Griffiths, G. , Beatty, M. , Gonzalez, S. M. , Bergman, D. A. , Hamilton, B. C. , Erickson, A. R. , Sokolove, J. , Robinson, W. H. , … O'Dell, J. R. (2014). Periodontitis and *Porphyromonas gingivalis* in patients with rheumatoid arthritis. Arthritis & Rhematology, 66, 1090–1100. 10.1002/art.38348. PMC4115329 24782175

[cre2391-bib-0022] Mohareri, M. , Mirhosseini, A. , Mehraban, S. , & Fazeli, B. (2018). Thromboangiitis obliterans episode: Autoimmune flare‐up or reinfection? Vascular Health and Risk Management, 14, 247–251. 30319267 10.2147/VHRM.S172047 PMC6168068

[cre2391-bib-0023] Nagase, K. (1975). Morphologic reaction of the arterial wall to local injuries caused by intraluminally applied endotoxin. Shinshu Medical Journal, 23, 253–275 (in Japanese).

[cre2391-bib-0024] Noack, B. , Genco, R. J. , Trevisan, M. , Grossi, S. , Zambon, J. J. , & Nardin, E. D. (2001). Periodontal infections contribute to elevated systemic C‐reactive protein level. Journal of Periodontology, 72, 1221–1227. 11577954 10.1902/jop.2000.72.9.1221

[cre2391-bib-0025] Offenbacher, S. , Barros, S. P. , & Beck, J. D. (2008). Rethinking periodontal inflammation. Journal of Periodontology, 79, 1577–1584. 18673013 10.1902/jop.2008.080220

[cre2391-bib-0026] Ohki, T. , Itabashi, Y. , Kohno, T. , Yoshizawa, A. , Nishikubo, S. , Watanabe, S. , Yamane, G. , & Ishikawa, K. (2012). Detection of periodontal bacteria in thrombi of patients with acute myocardial infarction by polymerase chain reaction. American Heart Journal, 163, 164–167. 22305832 10.1016/j.ahj.2011.10.012

[cre2391-bib-0027] Olin, J. W. (2000). Thromboangiitis obliterans (Buerger's disease). The New England Journal of Medicine, 343, 864–869. 10995867 10.1056/NEJM200009213431207

[cre2391-bib-0028] Penn, M. S. , & Chisolm, G. M. (1991). Relation between lipopolysaccharide‐induced endothelial cell injury and entry of macromolecules into the rat aorta in vivo. Circulation Research, 68, 1259–1269. 2018990 10.1161/01.res.68.5.1259

[cre2391-bib-0029] Rajakaruna, G. A. , Negi, M. , Uchida, K. , Sekine, M. , Furukawa, A. , Ito, T. , Kobayashi, D. , Suzuki, Y. , Akashi, T. , Umeda, M. , Meinzer, W. , Izumi, Y. , & Eishi, Y. (2018). Localization and density of *Porphyromanas gingivalis* and *Tannerella forsythia* in gingival and subgingival granulation tissues affected by chronic or aggressive periodontitis. Scientific Reports, 8, 9507. 10.1038/s4198-018-27766-7. 29934515 PMC6014976

[cre2391-bib-0030] Rudney, J. D. , Chen, R. , & Sedgewick, G. J. (2001). Intracellular *Actinobacillus actinomycetemcomitans* and *Porphyromonas gingivalis* in buccal epithelial cells collected from human subjects. Infection and Immunity, 69, 2700–2707. 11254637 10.1128/IAI.69.4.2700-2707.2001 PMC98209

[cre2391-bib-0031] Sandros, J. , Papapanou, P. , & Dahlen, G. (1993). *Porphyromonas gingivalis* invades oral epithelial cells in vitro. Journal of Periodontal Research, 28, 219–226. 8388449 10.1111/j.1600-0765.1993.tb01072.x

[cre2391-bib-0032] Seymour, G. J. , Ford, P. J. , Cullinan, M. P. , Leishman, S. , & Yamazaki, K. (2007). Relationship between periodontal infections and systemic disease. Clinical Microbiology and Infection, 13(Suppl. 4), 3–10. 17716290 10.1111/j.1469-0691.2007.01798.x

[cre2391-bib-0033] Shionoya, S. (1990). Etiology, pathology, clinical manifestation, diagnosis. In S. Shionoya (Ed.), Buerger's disease: Pathology, diagnosis and treatment (pp. 38–198). Nagoya, Japan: University of Nagoya Press.

[cre2391-bib-0034] Socransky, S. S. , & Haffajee, A. D. (2000). Dental biofilms: Difficult therapeutic targets. Periodontology, 28, 12–55. 10.1034/j.1600-0757.2002.280102.x 12013340

[cre2391-bib-0035] Sopori, S. (2002). Effects of cigarette smoke on the immune system. Nature Reviews Immunology, 2, 372–377. 10.1038/nri803 12033743

[cre2391-bib-0036] Suwatanapongched, P. , Surarit, R. , Srisatjaluk, R. , & Offenbacher, R. (2010). Translocation of *Porphyromanas gingivalis* infected monocytes and associated cellular response. Asian Pacific Journal of Allergy and Immunology, 28, 192–199. 21038790

[cre2391-bib-0037] Tanaka, K. (1998). Pathology and pathogenesis of Buerger's disease. International Journal of Cardiology, 66(Suppl. 1), S235–S242. 10.1016/s0167-5273(98)00174-0 9951825

[cre2391-bib-0038] Vergnes, J. N. , & Sixou, M. (2007). Preterm low birth weight and maternal periodontal status: A meta‐analysis. American Journal of Obstetrics and Gynecology, 196, 135.e1–135.e7. 17306654 10.1016/j.ajog.2006.09.028

[cre2391-bib-0039] Wessler, S. , Ming, S. C. , Gurewich, V. , & Freiman, D. G. (1961). A critical evaluation of thromboangiitis obliterans. The case against Buerger's disease. The New England Journal of Medicine, 262, 1149–1162. 10.1056/NEJM196006092622301 13844117

[cre2391-bib-0040] Wu, T. , Trevisan, M. , Genco, R. J. , Dorn, J. P. , Falkner, K. L. , & Sempos, C. T. (2000). Periodontal disease and risk of cerebrovascular disease. Archives of Internal Medicine, 160, 2749–2755. 11025784 10.1001/archinte.160.18.2749

[cre2391-bib-0041] Zimmer, S. , Steinmetz, M. , Asdonk, T. , Motz, I. , Coch, C. , Hartmann, E. , Barchet, W. , Wassmann, S. , Hartmann, G. , & Nickenig, G. (2011). Activation of endothelial toll‐like receptor 3 impairs endothelial function. Circulation Research, 108, 1358–1366. 21493895 10.1161/CIRCRESAHA.111.243246

